# Administration Timing and Efficacy of Tocilizumab in Patients With COVID-19 and Elevated IL-6

**DOI:** 10.3389/fmolb.2021.651662

**Published:** 2021-04-15

**Authors:** Pan Li, Zhengmao Lu, Qiang Li, Zhenmeng Wang, Yan Guo, Chen Cai, Shengyun Wang, Peng Liu, Xiaoping Su, Yi Huang, Yuchao Dong, Wenjuan Qiu, Yueming Ling, Lonny Yarmus, Fengming Luo, Li Zeng, Chong Bai, Wei Zhang

**Affiliations:** ^1^Department of Cardiology, Changhai Hospital, Second Military Medical University, Shanghai, China; ^2^Department of Infection Diseases No. 1, The Maternal and Child Health Hospital of Hubei Province, Wuhan, China; ^3^Department of Gastrointestinal Surgery, Changhai Hospital, Second Military Medical University, Shanghai, China; ^4^Department of Neurosurgery, Changhai Hospital, Second Military Medical University, Shanghai, China; ^5^Department of Anesthesia, Third Affiliated Hospital, Second Military Medical University, Shanghai, China; ^6^Department of Endocrinology, Changhai Hospital, Second Military Medical University, Shanghai, China; ^7^Department of Special Clinic, Changhai Hospital, Second Military Medical University, Shanghai, China; ^8^Department of Emergency and Critical Care Medicine, Changzheng Hospital, Second Military Medical University, Shanghai, China; ^9^Department of Colorectal Surgery, Changhai Hospital, Second Military Medical University, Shanghai, China; ^10^School of Basic Medicine, Wenzhou Medical University, Wenzhou, China; ^11^Department of Respiratory and Critical Care Medicine, Changhai Hospital, Second Military Medical University, Shanghai, China; ^12^Department of Cardiovascular ICU, Changhai Hospital, Second Military Medical University, Shanghai, China; ^13^Department of Clinical Laboratory Science of No. 910 Hospital of PLA Joint Support Force, Quanzhou, China; ^14^Division of Pulmonary and Critical Care, Johns Hopkins University School of Medicine, Baltimore, MD, United States; ^15^Department of Pulmonary and Critical Care Medicine, West China Hospital, Sichuan University, Chengdu, China; ^16^Department of Organ Transplantation, Changhai Hospital, Second Military Medical University, Shanghai, China

**Keywords:** cytokine storm, interleukin-6, SARS-CoV-2, tocilizumab (TCZ), coronavirus – COVID-19

## Abstract

**Background:**

Tocilizumab (TCZ), an interleukin-6 receptor antibody, has previously been used for treating patients with the coronavirus disease 2019 (COVID-19), but there is a lack of data regarding the administration timing of TCZ.

**Objectives:**

This study aimed to evaluate the timing and efficacy of TCZ in the treatment of patients with COVID-19.

**Methods:**

Laboratory-confirmed patients with COVID-19 with an elevated interleukin-6 (IL-6) level (>10 pg/ml) were offered TCZ intravenously for compassionate use. Clinical characteristics, laboratory tests, and chest imaging before and after the administration of TCZ were retrospectively analyzed.

**Results:**

A total of 58 consecutive patients who met the inclusion criteria and with no compliance to the exclusion criteria were included. Of these 58 patients, 39 patients received TCZ treatment, and 19 patients who declined TCZ treatment were used as the control cohort. In the TCZ-treatment group, 6 patients (15.4%) were in mild condition, 16 (41.0%) were in severe condition, and 17 (43.6%) were in critical condition. After TCZ treatment, the condition of 27 patients (69.2%) improved and 12 (30.8%) died. Compared with the improvement group, patients in the death group had higher baseline levels of IL-6 (*P* = 0.0191) and procalcitonin (PCT) (*P* = 0.0003) and lower lymphocyte percentage (LYM) (*P* = 0.0059). Patients receiving TCZ treatment had better prognoses than those without TCZ treatment (*P* = 0.0273). Furthermore, patients with a baseline IL-6 level of ≥100 pg/ml in the TCZ-treatment group had poorer clinical outcomes than those with an IL-6 level of <100 pg/ml (*P* = 0.0051).

**Conclusion:**

The administration of TCZ in an early stage of cytokine storm (IL-6 level < 100 pg/ml) may effectively improve the clinical prognosis of patients with COVID-19 by blocking the IL-6 signal pathway.

## Introduction

Ever since the coronavirus disease 2019 (COVID-19) outbreak in December 2019, the disease rapidly continues to spread and has been declared a global pandemic (Wang et al., 2020). According to the real-time statistics released by Johns Hopkins University, the COVID-19 pandemic has led to 77,107,760 confirmed cases with a death toll of 1,696,995 worldwide, as of December 21, 2020. Although the reported incidence rate of COVID-19 is currently lower than that of SARS-CoV and MERS-CoV, the mortality rate among patients with severe COVID-19 is up to 60% (Arabi et al., 2020). More worrisome is the fact that the mechanisms of the underlying pathogenicity of COVID-19 are thus far not fully illustrated. This, in part, has resulted in limited specific antiviral treatments available for COVID-19.

During the SARS-CoV-2 epidemic, it was discovered that the immuno-pathological processes were involved in lung injury (Kuiken et al., 2003). In the biopsy samples of patients with SARS-CoV-2, extensive lung damage revealed the accumulation of monocytes, macrophages, and neutrophils in the lungs, resulting in elevated lung cytokine/chemokine levels (Moore and June, 2020). The levels of serum proinflammatory cytokines and chemokines were also found to increase in patients with COVID-19, including tumor necrosis factor α (TNF-α), interferon-γ (IFN-γ)-induced protein 10 (IP-10), interleukin-6 (IL-6), IL-8, and IL-10 (Chen N. et al., 2020; Huang et al., 2020). Therefore, the clinical deterioration of COVID-19 may partly result from the immunopathology induced by a hypercytokinemia or cytokine storm. Of note is that a large number of studies have shown that the levels of IL-6 were significantly higher in patients with severe COVID-19 as compared to those in uncomplicated individuals (Chen G. et al., 2020; Ye et al., 2020). These findings suggest that IL-6 might play a vital role in the severe deterioration of some patients.

Tocilizumab [TCZ, (Roche Pharma (Schweiz) Ltd., S20171024)], a human monoclonal antibody directed against IL-6, has been commonly used in the therapy of rheumatoid arthritis (RA) (Biggioggero et al., 2018). Recent studies have reported the benefit of TCZ in severely ill patients with COVID-19 (Luo et al., 2020; Stone et al., 2020; Xu et al., 2020). However, to our knowledge, the appropriate timing of the administration of TCZ in patients with COVID-19 is unknown. Herein, this study evaluated the efficacy and timing of the intravenous administration of TCZ for compassionate use in the treatment of patients with COVID-19.

## Materials and Methods

### Study Design and Participants

This single-center retrospective cohort study was performed at the Guanggu district, the Maternal and Child Health Hospital of Hubei Province, China, which is a designated hospital for the treatment of patients with COVID-19. The study protocol was approved by the Medical Ethics Committee of the Maternal and Child Health Hospital of Hubei Province [FYGG (L)-2020-018].

From February 19, 2020 to April 7, 2020, consecutive patients with confirmed COVID-19 were evaluated for inclusion in this study. The inclusion criteria were as follows: (1) age ≥ 18 years; (2) nasal swab samples that were confirmed to be positive for SARS-CoV-2 nucleic acid by real-time reverse-transcriptase-PCR (RT-PCR) or for serum novel coronavirus-specific IgM and IgG antibodies; and (3) IL-6 level exceeding the upper limit of the normal value (>10 pg/ml). The exclusion criteria were as follows: (1) allergic reactions to TCZ or any adjuvant and (2) active inflammatory disease, such as hepatitis, tuberculosis, terminal tumor, or rheumatism immunity.

The diagnosis and severity stratification of COVID-19 were made based on the guidelines released by the National Health Commission of China (trial version 7) (National Health Commission, 2020). Patients with moderate or common type of COVID-19 having fever, dyspnea, and other symptoms with the manifestation of pneumonia seen in imaging data were diagnosed as mild cases; patients with respiratory frequency ≥ 30/min, blood oxygen saturation ≤ 93% at room air, or PaO_2_/FiO_2_ ratio ≤ 300 mmHg were defined as severe cases; and patients with respiratory failure requiring mechanical ventilation, shock, or other organ dysfunction/failure in addition to COVID-19 needing a stay in the intensive care unit (ICU) for treatment were diagnosed as critical cases. Comparison of the clinical characteristics among patients with COVID-19 treated with or without TCZ (Table 1) is a cohort study, while comparison between clinical characteristics of patients in the improvement group and in the death group after receiving TCZ (Table 2) is a case-control study.

**TABLE 1:** Clinical characteristics of the enrolled patients with coronavirus disease 2019 (COVID-19) treated with or without tocilizumab (TCZ).

	Total (*N* = 58)	TCZ-treatment group (*N* = 39)	Non-TCZ treatment group (*N* = 19)	*p*-value
**Age, y**	73.9 ± 12.7	74.7 ± 13.4	72.3 ± 11.3	0.2812
**Gender, n (%)**				0.5685
Male	37 (63.8)	26 (66.7)	11(57.9)	
Female	21 (36.2)	13 (33.3)	8 (42.1)	
**BMI**	24.1 ± 2.1	24.3 ± 2.1	23.6 ± 2.0	0.2027
**Type, n (%)**				0.2034
Mild	12 (20.7)	6 (15.4)	6 (31.6)	
Severe	20 (34.5)	16 (41.0)	4 (21.0)	
Critical	26 (44.8)	17 (43.6)	9 (47.4)	
**Symptoms, n (%)**				
Fever history	39 (67.2)	23 (59.0)	16 (84.2)	0.0756
Fever at admission (°C)	9 (15.5)	6 (15.4)	3 (15.8)	1.0000
Dyspnea	18 (31.0)	7 (18.0)	11 (57.9)	**0.0053**
Fatigue	30 (51.7)	18 (46.2)	12 (63.2)	0.2708
Muscle soreness	9 (15.5)	6 (15.4)	3 (15.8)	1.0000
Chills	8 (13.8)	4 (10.3)	4 (21.1)	0.4179
Cough	30 (51.7)	17 (43.6)	13 (68.4)	0.0973
Throat irritation	8 (13.8)	1 (2.6)	7 (36.8)	**0.0011**
**Presence of comorbidities, n (%)**				
Hypertension	32 (55.2)	18 (46.1)	14 (73.7)	0.0558
Diabetes	22 (37.9)	10 (25.6)	12 (63.2)	**0.0092**
CHD	18 (31.0)	10 (25.6)	8 (42.1)	0.2367
Cerebrovascular events	6 (10.3)	3 (7.7)	3 (15.8)	0.5894
COPD Renal insufficiency	7 (12.1) 5 (8.6)	4 (10.3) 2 (5.1)	3 (15.8) 3 (15.8)	0.6726 0.3179
Oxygen saturation%	92 (86–95)	90 (85.0–93.0)	92 (87–96)	0.6416
Systolic blood pressure (mmHg)	132 (120–155)	130 (120–155)	136 (125–156)	0.1587
Diastolic blood pressure (mmHg) Body temperature (°C) **Treatment**	78.5 (70–88) 36.4 (36.2–36.8)	78 (69–86) 36.4 (36.2–36.9)	86 (74–90) 36.5 (36.2–36.6)	0.0861 0.9934
Antiviral treatment, n (%)	37 (63.8)	30 (76.9)	11 (57.9)	0.2181
Antibiotic treatment, n (%)	42 (72.4)	31 (79.5)	11 (57.9)	0.1190
Oxygen therapy				0.5801
Low-flow oxygen	24 (41.4)	14 (35.9)	10 (52.6)	
High-flow oxygen Non-invasive ventilation Invasive ventilation	10 (17.2) 9 (15.5) 15 (25.9)	7 (18.0) 6 (15.4) 12 (30.8)	3 (15.8) 3 (15.8) 3 (15.8)	
Glucocorticoid, n (%)	22 (37.9)	21 (53.9)	5 (26.3)	0.0558
**Laboratory characteristics**				
WBC (×10^9^/L)	7.4 (5.3–11.6)	6.4 (4.1–11.0)	10.9 (6.9–12.3)	**0.0170**
PCT (ng/ml)	0.13 (0.07–0.61)	0.09 (0.05–0.28)	0.20 (0.10–0.81)	0.0721
LDH (U/L)	291 (218–343)	285.5 (212–343)	287.5 (234–312)	0.9203
Lymphocyte count (× 10^9^/L)	0.77 (0.45–1.02)	0.70 (0.44–0.93)	0.83 (0.53–1.36)	0.0635
Lymphocyte percentage (%)	8.7 (5.3–17.6)	9.7 (5.2–17.6)	7.6 (5.3–18.4)	0.9208
Hs-CRP (mg/L)	58.3 (24.4–116.8)	35.4 (9.1–92.8)	82.1 (25.5–101.9)	0.9406
IL-6 (pg/ml)	77.8 (34.1–302.0)	63.4 (36.4–129.7)	25.8 (29.8–82.6)	**0.0014**
D-dimer (mg/l)	3.7 (1.3–6.5)	2.9 (1.3–5.3)	4.3 (1.4–12.5)	0.3375

*Three methods have been used for testing: the Wilcoxon rank-sum test for continuous variables, the Fisher’s exact test for dichotomous variables, and the General Linear Model for categorical variables. All continuous variables were expressed as median (interquartile range [IQR]), except for age, which was represented as mean ± SD. CHD, coronary artery heart disease; COPD, chronic obstructive pulmonary disease; hs-CRP, high-sensitivity C-reactive protein; IL-6, interleukin-6; LDH, lactate dehydrogenase; PCT, procalcitonin; WBC, white blood cell. There was statistical difference in bold type.*

**TABLE 2:** Clinical characteristics of the patients with COVID-19 in the improvement group or the death group after receiving TCZ.

	Total (*N* = 39)	Improvement group (*N* = 27)	Death group (*N* = 12)	*p*-value
**Age, y**	74.7 ± 13.4	75.0 ± 12.3	73.8 ± 16.1	0.3413
**Gender, n (%)**				1.0000
Male	26 (66.7)	18 (66.7)	8 (66.7)	
Female	13 (33.3)	9 (33.3)	4 (33.3)	
**BMI**	24.3 ± 2.1	24.3 ± 2.3	24.3 ± 1.4	0.9747
**Type, n (%)**				0.0003
Mild	6 (15.4)	6 (22.2)	0	
Severe	16 (41.0)	15 (55.6)	1 (8.3)	
Critical	17 (43.6)	6 (22.2)	11 (91.7)	
**Symptoms, n (%)**				
Fever History	23 (59.0)	19 (70.4)	4 (33.3)	**0.0407**
Fever at admission (°C)	6 (15.4)	6 (22.2)	0	0.1508
Dyspnea	7 (18.0)	4 (14.8)	3 (25.0)	0.6536
Fatigue	18 (46.2)	12 (44.4)	6 (50.0)	1.0000
Muscle soreness	6 (15.4)	4 (14.8)	2 (16.7)	1.0000
Chills	4 (10.3)	3 (11.1)	1 (8.3)	1.0000
Cough	17 (43.6)	10 (37.0)	7 (58.3)	0.2994
Throat irritation	1 (2.6)	1 (3.7)	0	1.0000
**Presence of comorbidities, n (%)**				
Hypertension	18 (46.1)	14 (51.9)	4 (33.3)	0.3221
Diabetes	10 (25.6)	7 (25.9)	3 (25.0)	1.0000
CHD	10 (25.6)	7 (25.9)	3 (25.0)	1.0000
Cerebrovascular events	3 (7.7)	3 (11.1)	0	0.5391
COPD Renal insufficiency	4 (10.3) 2 (5.1)	4 (14.8) 1 (3.7)	0 1 (8.3)	0.2916 0.5263
Oxygen saturation%	90 (85.0–93.0)	92 (88.0–95.0)	86.5 (84.5–92.5)	0.0523
Systolic blood pressure (mmHg)	130 (120–155)	130 (120–155)	128 (120–154)	0.9392
Diastolic blood pressure (mmHg)	78 (69–86) 36.4 (36.2–36.9)	78 (69–84) 36.4 (36.2–36.9)	80 (66.5–88.5) 36.25 (36.15–36.85)	0.7839 0.2983
Body temperature (°C)				
**Treatment**				
Antiviral treatment, n (%)	30 (76.9)	21 (77.8)	9 (75.0)	1.0000
Antibiotic treatment, n (%)	31 (79.5)	20 (74.1)	11 (91.7)	0.3938
Oxygen therapy				0.0004
Low-flow oxygen	14 (35.9)	14 (51.9)	0	
High-flow oxygen Non-invasive ventilation Invasive ventilation	7 (18.0) 6 (15.4) 12 (30.8)	6 (22.2) 3 (11.1) 4 (14.8)	1 (8.3) 3 (25.0) 8 (66.7)	
Glucocorticoid, n (%)	21 (53.9)	11 (40.7)	10 (83.3)	**0.0180**
**Laboratory characteristics**				
WBC (× 10^9^/L)	6.4 (4.1–11.0)	6.3 (4.1–8.8)	8.55 (4.9–12.3)	0.3377
PCT (ng/ml)	0.09 (0.05–0.28)	0.07 (0.05–0.11)	0.79 (0.16–1.88)	**0.0003**
LDH (U/L)	285.5 (212–343)	264.5 (192–310)	499.5 (412–614.5)	**0.0056**
Lymphocyte count (× 10^9^/L)	0.7 (0.44–0.93)	0.71 (0.44–0.93)	0.65 (0.49–0.89)	0.9515
Lymphocyte percentage (%)	9.7 (5.2–17.6)	14.6 (6.8–20.5)	6.2 (3.0–7.1)	**0.0059**
Hs-CRP (mg/L)	35.4 (9.1–92.8)	24.4 (9.1–87.2)	54.7 (23.9–112.7)	0.1709
IL-6 (pg/ml)	63.4 (36.4–129.7)	53.2 (33.8–91.4)	140.4 (69.9–316.5)	**0.0191**
D-dimer (mg/L)	2.9 (1.3–5.3)	2.7 (1.0–5.1)	3.5 (1.6–6.5)	0.3381

*The tests to determine the laboratory characteristics presented in Table 2 were conducted at baseline. Three methods have been used for testing: the Wilcoxon rank-sum test for continuous variables, the Fisher’s exact test for dichotomous variables, and the General Linear Model for categorical variables. All continuous variables were expressed as median (interquartile range [IQR]), except for age, which was represented as mean ± SD. CHD, coronary artery heart disease; COPD, chronic obstructive pulmonary disease; hs-CRP, high-sensitivity C-reactive protein; IL-6, interleukin-6; LDH, lactate dehydrogenase; PCT, procalcitonin; WBC, white blood cell. There was statistical difference in bold type.*

### TCZ Treatment and Observation

Tocilizumab of 4–8 mg/kg, to a maximum dose of 800 mg, was intravenously administered once within 60 min of patients meeting the inclusion criteria. For patients who still had fever 24 h after the administration of the first dose, TCZ was readministered with an interval of ≥12 h between the two administrations. Other routine treatments such as antiviral, antibiotic, corticosteroid treatment, and oxygen therapy were continued as per physician discretion.

The clinical data including medical history, clinical symptoms, comorbidities, imaging data, treatment measures, and clinical outcomes were collected and analyzed. Laboratory examinations, including complete blood count, liver and renal function, IL-6 levels, high-sensitive C-reactive protein (hs-CRP), procalcitonin (PCT), myocardial enzymes, and coagulation analysis, were collected and analyzed. Elevated IL-6 level was defined to be >10 pg/ml (Pylon immunoassay system); elevated hs-CRP was defined to be >10 mg/L; and elevated PCT was defined to be >0.05 ng/ml, as per the specifications of the manufacturer. The nasopharyngeal swab test for SARS-CoV-2 (Shanghai ZJ Bio-Tech Co., Ltd.) was performed when the patients were admitted to the isolation ward, and it was repeated twice with an interval of at least 24 h before discharge using RT-PCR.

### Statistical Analysis

All variables were expressed as the mean ± SD or median (interquartile range [IQR]) or number (%). The Wilcoxon rank-sum test was used for continuous variables, the Fisher’s exact test was used for dichotomous variables, and the general linear model was used for categorical variables. The Wilcoxon signed-rank test was used for paired data analysis before and after treatment with TCZ for both patients in the improvement group and those in the death group. The difference of median IL-6 and of median hs-CRP categorized by the outcomes and the difference of median lymphocyte (LYM) count categorized by the baseline IL-6 level were tested by using the Wilcoxon rank-sum test. The Kaplan–Meier methodology was used to estimate the probability of survival. The survival rate was compared between the groups treated with and without TCZ (IL-6, LYM%, hs-CRP, and PCT) using the log-rank test at the two-sided significance level. The multivariable Cox proportional-hazards model analysis was used to explore the role of TCZ in clinical outcomes when adjusted for clinical covariates, including age, body mass index (BMI), diabetes, and glucocorticoid. Statistical analyses were performed using the SAS Studio version 3.7 software. A *P*-value of < 0.05 was considered statistically significant.

## Results

### Demographic Characteristics

**FIGURE 1:**
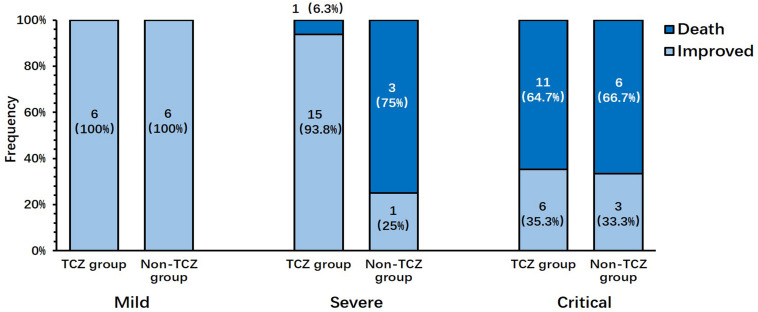
Distribution of disease severity in patients who improved or died after treatment with or without tocilizumab.

Between February 19, 2020 and April 7, 2020, a total of 58 patients met the inclusion criteria, and no patients who met the exclusion criteria (Table 1) were screened. Of these 58 patients, 39 patients signed informed consent and received TCZ treatment and 19 patients, who declined TCZ treatment, were used as the control cohort. The majority of patients (37, 63.8%) were male, with a mean age of 73.9 ± 12.7 years. There were 12 (20.7%) mild cases, 20 (34.5%) severe cases, and 26 (44.8%) critical cases. A total of 39 consecutive patients receiving TCZ treatment were divided into two groups: the improvement group (27 cases, 69.2%) and the death group (12 cases, 30.8%; Table 2). Six (22.2%) mild cases, 15 (55.6%) severe cases, and 6 (22.2%) critical cases were observed in the improvement group; meanwhile, 1 (8.3%) severe case and 11 (91.7%) critical cases were present in the death group; no patients with mild cases were present in the death group (*P* = 0.0003). The majority of patients (97.4%) received TCZ once. One patient (2.6%) received a second dose with the same dosage due to the occurrence of fever within 12 h. In contrast, patients in the non-TCZ treatment group had a higher death rate trend (9/19, 47.4%), especially those in the severe case group (3/4, 75%; Figure 1).

### Clinical Presentations and Safety

A total of 67.2% of the patients presented with fever at the onset, followed by fatigue (51.7%), dry cough (51.7%), dyspnea (31.0%), muscle soreness (15.5%), and chills (13.8%). Among patients treated with TCZ, no significant differences were found between the improvement group and the death group in terms of blood oxygen saturation, blood pressure, and body temperature at baseline. The most common comorbidity was hypertension (55.2%), followed by diabetes (37.0%), coronary heart disease (31.0%), tumor (20.7%), and chronic obstructive pulmonary disease (COPD) (12.1%). There was no difference in the incidence of each comorbidity between the improvement group and the death group (all *P* > 0.05, Table 1).

During the hospital stay, 83.3% of patients in the death group and 40.7% of patients in the improvement group received corticosteroids (*P* = 0.018, Table 2), but there was no difference in the proportion of corticosteroids administered between the TCZ- and the non-TCZ treatment groups. Patients in the death group received a higher rate of invasive ventilation than those in the improvement group (*P* = 0.0004, Table 2). No differences were observed between the death group and the improvement group on receiving other treatments. No adverse effects, such as rash, nausea, thrombocytopenia, neutropenia, and abnormal liver and kidney function, were observed after treatment with TCZ.

### Laboratory Examinations

At baseline, patients in the death group had a lower percentage of lymphocytes (6.2 [3.0–7.1]) in comparison to those in the improvement group (14.6 [6.8–20.5], *P* = 0.0059). The values of inflammatory markers including PCT and IL-6 before the use of TCZ were significantly higher in the death group than in the improvement group (PCT: 0.79 [0.16–1.9] vs. 0.07 [0.05-0.11], *P* = 0.0003; IL-6: 140.4 [69.9–316.5] vs. 53.2 [33.8–91.4], *P* = 0.0191). After the treatment with TCZ, the level of lactate dehydrogenase (LDH), hs-CRP, IL-6, and D-dimer decreased significantly (all *P* < 0.05) in the improvement group, while the lymphocyte count and the percentage of lymphocytes increased (all *P* < 0.05, Table 3). In contrast, in the death group, there was no significant improvement in the laboratory indexes after the use of TCZ.

**TABLE 3:** Laboratory tests before and after the administration of tocilizumab in the improvement group and the death group.

	Improvement group (*N* = 27)		*p*-value	Death group (*N* = 12)		*p*-value
			
	Before TCZ	After TCZ		Before TCZ	After TCZ	
WBC (× 10^9^/L)	6.3 (4.1–8.8)	4.8 (3.9–6.2)	0.0746	8.55 (4.9–12.3)	9.8 (4.6–14.0)	0.8311
PCT (ng/ml)	0.07 (0.05–0.11)	0.05 (0.04–0.06)	0.3088	0.79 (0.16–1.9)	1.75 (0.44–5.5)	0.3750
LDH (U/L)	264.5 (192–310)	219 (193–234)	**0.0096**	499 (412–614.5)	537 (488.5–626.5)	0.8750
Lymphocyte count (× 10^9^/L)	0.71 (0.44–0.93)	1.3 (1.1–1.5)	**<0.0001**	0.65 (0.49–0.89)	0.265 (0.12–0.81)	**0.0269**
Lymphocyte percentage (%)	14.6 (6.8–20.5)	24.6 (15.9–29.9)	**<0.0001**	6.15 (3.00–7.05)	7.05 (3.4–11.4)	1.0000
Hs-CRP (mg/L)	24.4 (9.07–87.2)	1.42 (0.56–5.06)	**<0.0001**	54.7 (23.9–112.2)	20.67 (9.5–125.9)	0.7910
IL-6 (pg/ml)	53.2 (33.8–91.4)	110.5 (16.0–3327.1)	**0.0132**	140.4 (69.9–316.5)	1216 (584.9–3504.0)	**0.0059**
D-dimer (mg/L)	2.7 (1.0–5.1)	1.2 (0.73–2.7)	**<0.0001**	3.51 (1.6–6.5)	10.17 (2.8–21.9)	**0.0371**
Oxygen saturation%	92 (88–95)	98 (97–99)	**<0.0001**	86.5 (84.5–92.5)	85 (80–92.5)	0.6240

*CHD, coronary artery heart disease; COPD, chronic obstructive pulmonary disease; hs-CRP, high-sensitive C-reactive protein; IL-6, interleukin-6; LDH, lactate dehydrogenase; PCT, procalcitonin; TCZ, tocilizumab; WBC, white blood cell. There was statistical difference in bold type.*

**FIGURE 2:**
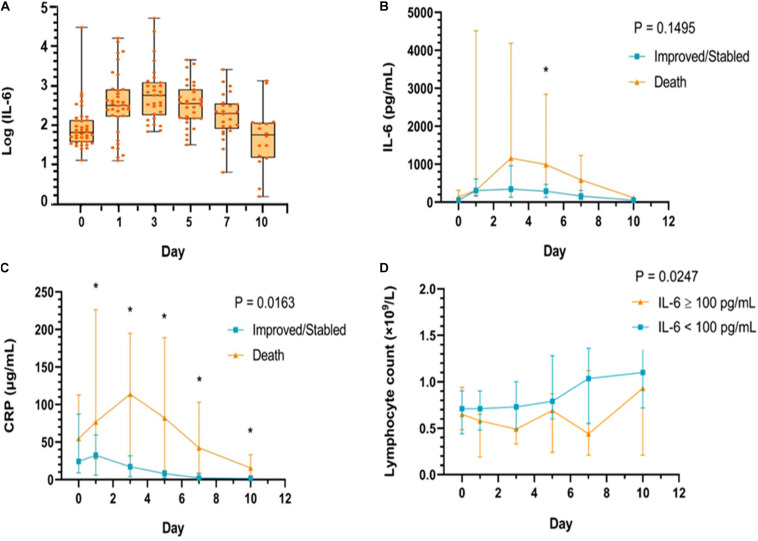
The changing trend of IL-6 levels before and after tocilizumab treatment **(A)**. The change of IL-6 **(B)** and hs-CRP **(C)** levels, respectively, during the tocilizumab treatment categorized by outcomes (improvement and death) at baseline, day 1, day 3, day 5, day 7, and day 10; **(D)** The change of lymphocytes (LYM) during the tocilizumab treatment categorized by baseline IL-6 level (using median value 100 pg/ml as cut-off value) at baseline, day 1, day 3, day 5, day 7, and day 10. *P < 0.05 compared with the improvement and death group.

The level of IL-6 observed after receiving TCZ treatment is shown in Figure 2A. From baseline, the level of IL-6 tended to increase on the first day after the use of TCZ and reached a peak on the third day, and then, it started to decrease on the fifth day and returned to baseline on the tenth day. However, the overall trend of the levels of IL-6 between patients in the improved group and those in the death group after TCZ treatment was not associated with the clinical outcome (*P* = 0.1495). Also, there was no significant difference in the level of IL-6 between the groups at each time point (All *P* > 0.05). After TCZ treatment, there was also a significant difference in hs-CRP levels within 10 days between patients in the improved and patients in the death group (*P* = 0.0163), indicating that the overall trend of the levels of hs-CRP after TCZ treatment was associated with the clinical outcome (Figure 2C), whereas the overall trend of the levels of IL-6 was not (Figure 2B).

**FIGURE 3:**
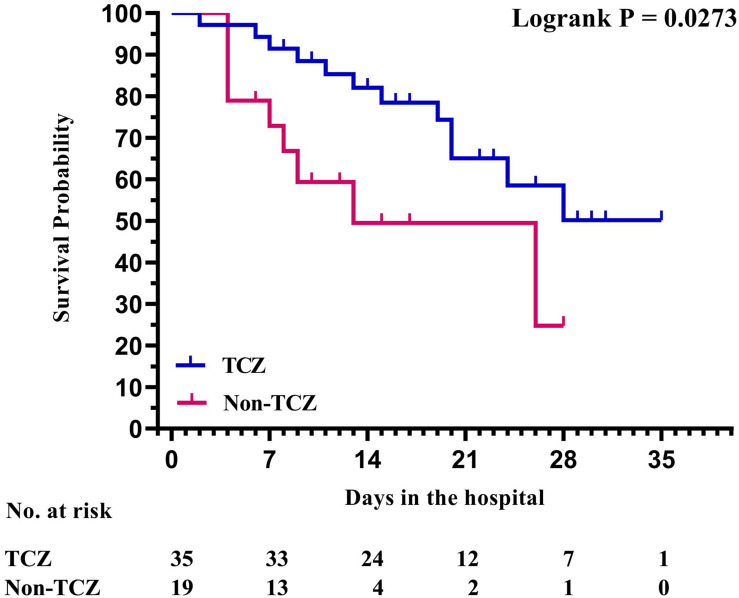
Survival analysis with Kaplan–Meier curves between patients with COVID-19 treated with or without tocilizumab.

**FIGURE 4:**
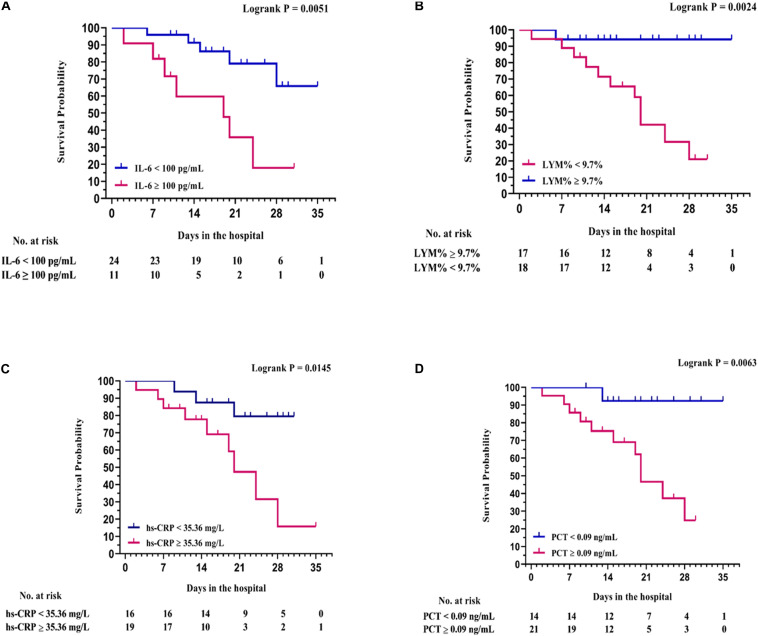
Survival analysis with Kaplan–Meier curves between baseline laboratory examination groups. **(A)** IL-6, using the median value 100 pg/ml as cut-off value; **(B)** LYM%, using the median value of 9.7% as cut-off value; **(C)** hs-CRP, using the median value of 35.36 mg/L as cut-off value; **(D)** PCT, using the median value of 0.09 ng/ml as cut-off value.

In addition, based on the cut-off value of 100 pg/ml for IL-6 at baseline (10 times the upper limit of normal; e.g., 10 pg/ml), all patients in the study were divided into two groups: patients with an IL-6 level of ≥100 pg/ml and patients with an IL-6 level of <100 pg/ml. The elevated trend of the level of lymphocytes was found in both groups after receiving TCZ, and there was a significant difference between the two groups (*P* = 0.0247, Figure 2D). The Kaplan–Meier curves showed that the survival rate of patients in the TCZ treatment group was significantly higher than that of patients in the non-TCZ treatment group (*P* = 0.0273, Figure 3). The multivariable Cox proportional-hazards model showed that TCZ was significantly associated with a lower risk of death (HR 0.383; 95% CI, 0.157 to 0.935; *P* = 0.035; Supplementary Figure 2). Furthermore, for patients receiving TCZ, the survival rate of patients with an IL-6 baseline level ≥ 100 pg/ml was worse than that of the patients with an IL-6 level < 100 pg/Ml; similar findings were also observed in patients with LYM% < 9.7, hs-CRP > 35.36 mg/L, and PCT ≥ 0.09 ng/ml (Figure 4).

## Discussion

This is the first study assessing the optimal timing of intravenous TCZ in patients with COVID-19. The preliminary data presented in this paper showed that, among the 39 patients with COVID-19 who received TCZ treatment, the majority of patients (27, 69.2%) either improved or stabilized without obvious side effects. TCZ, in this cohort, exhibited a good clinical efficacy and safety profile. Twelve patients (30.8%) in this cohort that died after TCZ therapy during hospitalization showed significantly higher IL-6, PCT, and LDH levels and lower lymphocyte percentages at baseline than those with clinical improvement. Patients receiving TCZ treatment had more survival probability than those that did not receive TCZ treatment. IL-6 levels ≥ 100 pg/ml, hs-CRP levels ≥ 35.56 mg/L, PCT levels ≥ 0.09 ng/ml, and LYM% < 9.7% were closely associated with poor prognoses after TCZ therapy. These findings collectively suggest that TCZ may be considered in the early stages of a cytokine storm (IL-6 level < 100 pg/ml).

Lymphopenia is a common feature in patients with severe COVID-19 and is also a predictor of poor prognosis (Yang et al., 2020). Specifically, the number of T cells, B cells, and natural killer cells decreased dramatically, while the number of neutrophils increased significantly. A cytokine storm in coronavirus disease results in the formation of cytokines storm, indicating an increase in proinflammatory cytokines, such as IL-6, IL-1β, IL-8, and IL-17; granulocyte colony-stimulating factor (G-CSF); granulocyte-macrophage colony-stimulating factor (GM-CSF); monocyte chemoattractant protein 1 (MCP-1); macrophage inflammatory protein-1α (MIP-1α); and TNF-α (Cheung et al., 2005; Law et al., 2005). The abundant release of inflammatory cytokines may mediate acute respiratory distress syndrome (ARDS), leading to inflammatory cell infiltration and diffused alveolar damage within a short time. The cytokine storm can then cause further severe inflammatory damage to multiple organs, including the heart, liver, and kidney. The complex immune process induced by the SARS-CoV-2 could explain why some patients deteriorate suddenly at a later stage or during the recovery period (Special Expert Group for Control of the Epidemic of Novel Coronavirus Pneumonia of the Chinese Preventive Medicine Association, 2020).

Previous studies had reported that IL-6 positively correlated with the severity of COVID-19, with higher levels of IL-6 present in patients with severe and critical COVID-19 than those present in patients with common illness (Chen L. et al., 2020). IL-6 has a prominent proinflammatory effect through binding to the membrane-bound IL-6 receptor (mIL-6R) in a complex with gp130; on the other hand, IL-6-related downstream signal path is mediated by JAKs and STAT3 (Kang et al., 2019). An IL-6 antagonist may effectively reduce mortality in autoimmune diseases by inhibiting cytokine storms (Jones et al., 2011; Wolf et al., 2014). There is pre-existing evidence which suggests that TCZ can improve the clinical outcome in patients with severe and critical COVID-19 (Luo et al., 2020; Price et al., 2020; Toniati et al., 2020; Xu et al., 2020). In the current study, 27 patients (69.2%) who received TCZ had an obvious improvement of clinical symptoms, and the release of an *in vivo* inflammatory response without toxicity or adverse reactions, such as peripheral blood leukocyte decrease or hepatic and renal function abnormity, was observed. The outcomes in the patient cohort in this study suggest that TCZ may be an effective and safe treatment option in patients with COVID-19 with cytokine storms.

Cytokine storm is a highly complex condition, showing an excessive immune response to external stimuli (Davidson et al., 2015). Once a cytokine storm forms during COVID-19 infection and reaches its peak, it may be refractory to treatment. Therefore, the timing of the administration of TCZ is an important factor that affected the prognosis of patients with severe and critical illnesses. In the current study, the rate of death (30.8%) after TCZ treatment is higher than previously reported data. The possible reasons are as follows: (1) the rate of critically ill patients with COVID-19 in the present study is almost 50%, which is higher than that in previously published reports (Xu et al., 2020: 19.0%; Luo et al., 2020: 26.7%; Toniati et al., 2020: 20%; Price et al., 2020: 25%); (2) patients in this study seemed to have a lower baseline oxygen saturation (median SpO_2_ 90%) than those in the previous studies (SpO_2_ range 91–99%) (Xu et al., 2020), suggesting that patients included in this study may be more seriously ill; (3) about 64.7% of critically ill patients in this study died after TCZ treatment, which suggests that later administration of TCZ in critically ill patients with evidence of an existing cytokine storm may be ineffective. Remarkably, of the 12 patients who died during the TCZ treatment, 58.3% patients had higher baseline levels of IL-6 than the cutoff value of 100 pg/ml; whereas, in the improvement group, only 18.5% patients had IL-6 levels above this cutoff value.

Compared to the TCZ treatment group, patients in the non-TCZ treatment group had a numerically higher death rate (47.4% vs. 30.8%) and were closely associated with a lower survival rate (*P* = 0.0273), indicating the improvement effect of TCZ on the prognoses of patients with COVID-19. In particular, a significant improvement was observed in the majority of severe cases (55.6%) receiving TCZ treatment, whereas the improvement rate of severe cases was only 10% in the non-TCZ group. More importantly, the Kaplan–Meier survival curves showed that the baseline IL-6 level of ≥100 pg/ml was closely associated with poor prognosis after TCZ treatment whereas at different cutoff values for the IL-6 baseline level, including IL-6 baseline level ≥ 30 pg/ml vs. < 30 pg/ml (*P* = 0.454) and IL-6 baseline level ≥ 50 pg/ml vs. < 50 pg/ml (*P* = 0.076), both of them have no significant correlation with clinical prognosis (Supplementary Figure 1). Accordingly, we recommend that the suitable timing to administer TCZ in patients with COVID-19 is in the early stage of the cytokine storm and, in particular, when the baseline level of IL-6 is less than 100 pg/ml. The early use of TCZ may effectively prevent excessive inflammatory injury and improve the outcome of severely ill patients with COVID-19.

At present, there is no clear conclusion about the time of and dose of administration of TCZ. In this study, based on the guidelines released by the National Health Commission of China, the majority of patients with COVID-19 (97.4%) received TCZ (4–8 mg/kg) once and one patient (2.6%) received a second dose due to the occurrence of fever. The results presented in this paper showed that the patients receiving TCZ treatment had better prognoses than those that did not receive TCZ treatment. Similarly, Xu et al. (2020) reported that TCZ (intravenous infusion of 400 mg) is an effective treatment for severely ill patients with COVID-19. Toniati et al. (2020) treated 100 patients with COVID-19 with TCZ at a dosage of 8 mg/kg (two consecutive intravenous infusions 12 h apart) and found that the treatment was associated with a clinical improvement in more than three-quarters of the patients. Moreover, Luo et al. (2020) suggested that repeated dose of the TCZ is recommended for critically ill patients with COVID-19 with elevated IL-6 levels. However, controversy remains as to whether the use of TCZ improves survival, shortens hospital stay, or reduces the need for mechanical ventilators. A randomized controlled study reported by Salama et al. (2021) demonstrated that TCZ reduced the likelihood of progression to mechanical ventilation or death by day 28 efficiently but that it did not improve the incidence of death from any cause. It appears that the timing of administration of TCZ is a critical factor that affects the prognosis.

The present study has several limitations. Previous data (Zhang et al., 2020) showed that D-dimer on admission could effectively predict the in-hospital mortality of patients with COVID-19. Although the improvement group had a numerically higher D-dimer level at baseline than that of the death group, no statistical difference was observed between the two groups, which may be due to the limited sample size in this study. Moreover, the results of a prospective meta-analysis (Sterne et al., 2020) showed that the administration of systemic corticosteroids among critically ill patients with COVID-19, compared with usual care or placebo, was associated with lower 28-day all-cause mortality. However, in the present study, there was no statistical difference in the use of corticosteroids between the improvement group and the death group after receiving TCZ. This study was conducted in the early stages of the COVID-19 epidemic, and there is a lack of clinical experience in the application of corticosteroids. We believe that the possible reason for the inconsistency of the results presented in this study compared with the reported data may be the different timing and dosages of glucocorticoids among the patients, as well as the limited sample size. Because this is a retrospective study, we did not compare the different dosages and the timing of administration of TCZ prospectively in this study. Therefore, the clinical practice value of the cutoff points of IL-6 in predicting the timing of TCZ administration needs further evaluation in future randomized controlled trials with large sample sizes and long-term clinical follow-up.

## Conclusion

This study suggests that TCZ may help to improve the prognosis of patients with COVID-19 with the baseline IL-6 level below the cutoff value of 100 pg/ml with an acceptable safety profile. Further clinical trials are required to confirm the observed findings and evaluate the efficacy of TCZ and its scope of clinical application and timing.

## Data Availability Statement

The raw data supporting the conclusions of this article will be made available by the authors, without undue reservation.

## Ethics Statement

The studies involving human participants were reviewed and approved by the study protocol was approved by the Medical Ethics Committee of the Maternal and Child Health Hospital of Hubei Province [FYGG (L)-2020-018]. The patients/participants provided their written informed consent to participate in this study.

## Author Contributions

PaL: data collection and writing. ZL: data collection and analysis. QL: data interpretation and analysis. ZW and WQ: literature search. YG and LY: manuscript preparation. CC: preparation of figures. SW: preparation of tables. PeL and YD: data interpretation. YH: literature search. YL: data collection. LZ: statistical analysis. CB: data analysis. WZ: study design and writing the manuscript. All authors: contributed to the article and approved the submitted version.

## Conflict of Interest

The authors declare that the research was conducted in the absence of any commercial or financial relationships that could be construed as a potential conflict of interest.

## Supplementary Material

Supplementary Figure 1 Survival analysis with Kaplan–Meier curves between baseline laboratory examination groups. **(A)** Il-6, using the median value of 30 pg/ml as cut-off value; **(B)** Il-6, using the median value of 50 pg/ml as cut-off value.

Supplementary Figure 2 Multivariable Cox proportional-hazards model analysis used to explore the role of tocilizumab on clinical outcomes when adjusted for clinical covariates, including age, Bmi, diabetes, and administration of glucocorticoids.
