# Supplementary figures and images for: Administration Timing and Efficacy of Tocilizumab in Patients With COVID-19 and Elevated IL-6
Source: Front Mol Biosci. 2021 Apr 15;8:651662. doi: 10.3389/fmolb.2021.651662 (PMC8084410; doi:10.3389/fmolb.2021.651662)

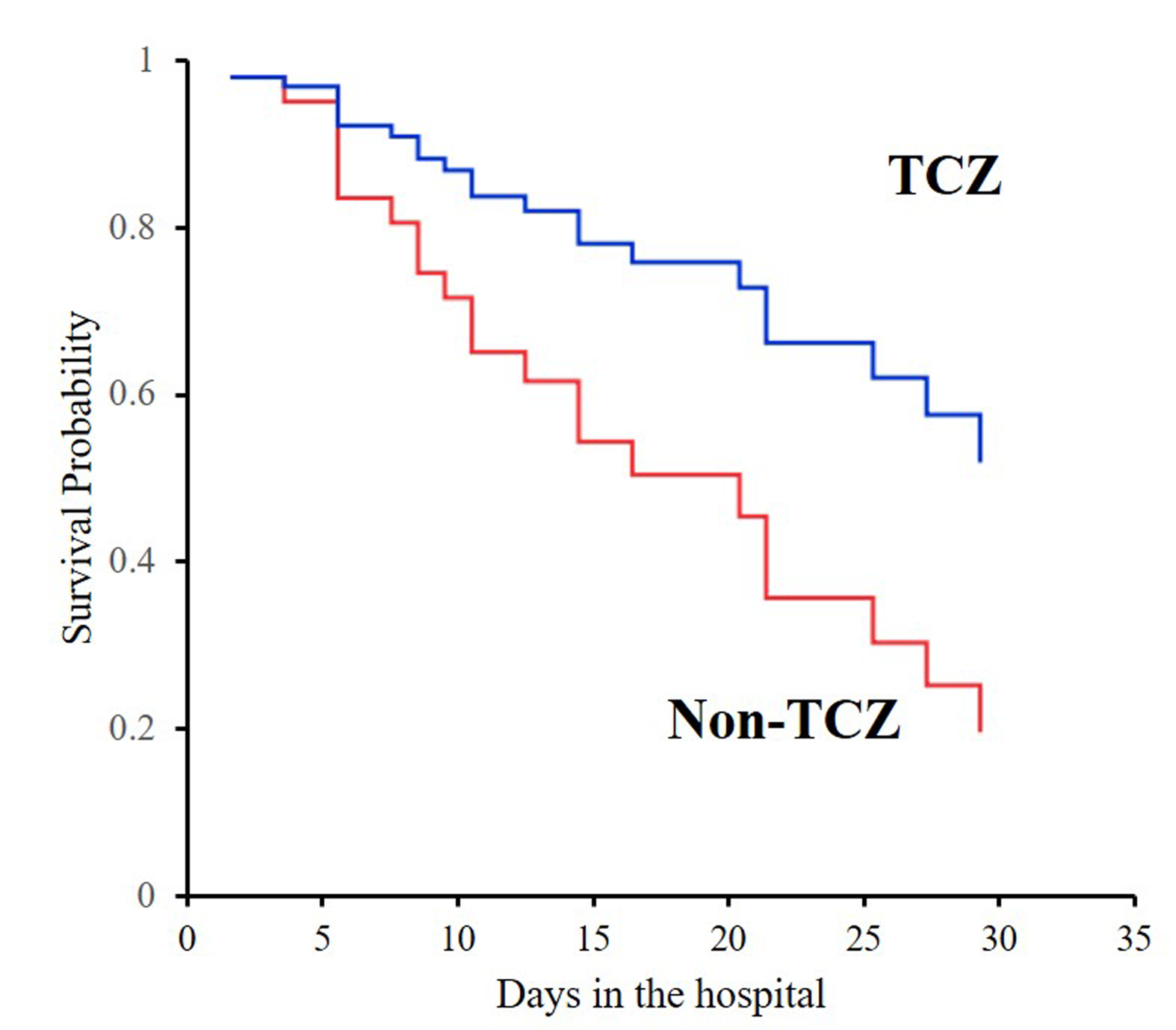

Supplement: Supplementary Figure 1 — Survival analysis with Kaplan–Meier curves between baseline laboratory examination groups. (A) Il-6, using the median value of 30 pg/ml as cut-off value; (B) Il-6, using the median value of 50 pg/ml as cut-off value. [file Image_1.tif]

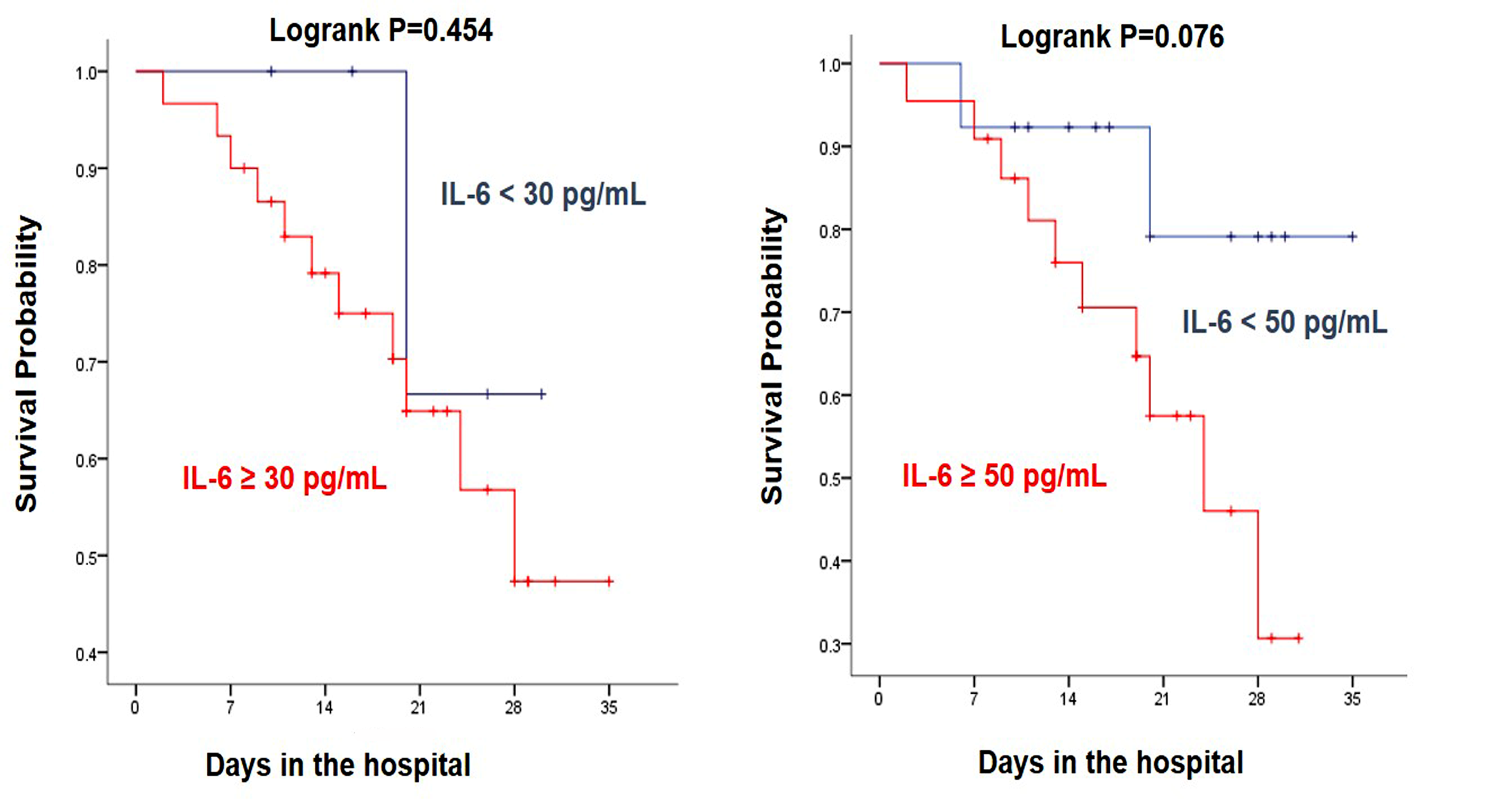

Supplement: Supplementary Figure 2 — Multivariable Cox proportional-hazards model analysis used to explore the role of tocilizumab on clinical outcomes when adjusted for clinical covariates, including age, Bmi, diabetes, and administration of glucocorticoids. [file Image_2.tif]
